# Pan-cancer analyses reveal multi-omics and clinical characteristics of RIO kinase 2 in cancer

**DOI:** 10.3389/fchem.2022.1024670

**Published:** 2022-11-28

**Authors:** Kexin Li, Jiahua Zou, Haizhao Yan, Yuqing Li, Man-Mei Li, Zhong Liu

**Affiliations:** ^1^ Guangdong Provincial Key Laboratory of Bioengineering Medicine, National Engineering Research Center of Genetic Medicine, Institute of Biomedicine, College of Life Science and Technology, Jinan University, Guangzhou, China; ^2^ CAS Key Laboratory of Regenerative Biology, Guangdong Provincial Key Laboratory of Stem Cell and Regenerative Medicine, Guangzhou Institutes of Biomedicine and Health, Chinese Academy of Sciences, Guangzhou, China; ^3^ International Cooperative Laboratory of Traditional Chinese Medicine Modernization and Innovative Drug Development of Chinese Ministry of Education (MOE), School of Pharmacy, Jinan University, Guangzhou, China

**Keywords:** RIO kinase 2, pan-cancer, mutation, methylation, phosphorylation, immune cells infiltration

## Abstract

RIO kinase 2 has emerged as a critical kinase for ribosome maturation, and recently it has also been found to play a fundamental role in cancer, being involved in the occurrence and progression of glioblastoma, liver cancer, prostate cancer, non-small cell lung cancer, and acute myeloid leukemia. However, our knowledge in this regard is fragmented and limited and it is difficult to determine the exact role of RIO kinase 2 in tumors. Here, we conducted an integrated pan-cancer analysis comprising 33 cancer-types to determine the function of RIO kinase 2 in malignancies. The results show that RIO kinase 2 is highly expressed in all types of cancer and is significantly associated with tumor survival, metastasis, and immune cell infiltration. Moreover, RIO kinase 2 alteration *via* DNA methylation, and protein phosphorylation are involved in tumorigenesis. In summary, RIO kinase two serves as a promising target for the identification of cancer and increases our understanding of tumorigenesis and cancer progression and enhancing the ultimate goal of improved treatment for these diseases.

## Introduction

RIO kinases (RIOK) belong to an evolutionarily conserved family of atypical kinases found in eukaryotes and archaea. There are three members of the RIO kinase family (RIOK1, RIOK2, and RIOK3), all of which are characterized by the inclusion of a unique RIO kinase domain (Yuan et al., 2014). The RIO domain is structurally homologous to the eukaryotic serine-threonine protein kinase domain, but it lacks classical activation and substrate-binding loops (Yuan et al., 2014). Kinase activity in humans is controlled by several mechanisms, including gene expression, alteration, epigenetic modification, and post-translational modification. RIOK has been demonstrated to exist in and be related to the maturation of pre-40S ribosomes (Leonard et al., 1998), a process in which the structure and function of RIOK2 have received a great deal of attention. Here, RIOK2 functions as a trans-acting factor that shepherds the last steps of maturation of the 40S ribosome subunit (Ameismeier et al., 2020). However, a dysregulation of kinase activity programs can cause a broad range of human diseases.

Recently, RIOK2 dysregulation has been implicated in the progression of various human cancers, including that of non-small cell lung cancer (Liu et al., 2016; Liu et al., 2018), glioblastoma (Read et al., 2013; Song et al., 2020), hepatocellular carcinoma cells (Delman et al., 2019), acute myeloid leukemia (Messling et al., 2021) and prostate cancer8. Despite its involvement with the onset and evolution of tumor processes, RIOK2 remains poorly understood in this context.

Evidence has accumulated that RIOK2 is connected with diverse cellular processes in cancer development and progression, such as cellular proliferation, migration, invasion, and apoptosis (Liu et al., 2016; Liu et al., 2018; Delman et al., 2019; Mohamed et al., 2018; Read et al., 2013; Song et al., 2020) For example, RIOK2 promotes glioma cell migration and invasion through epithelial–mesenchymal transition (Ameismeier et al., 2020). The association of RIOK2 with bystin-like protein and mechanistic target of rapamycin kinase promotes tumor cell growth and survival through activation of AKT signaling in gliomas (Gao et al., 2021). ERGi-USU, a high affinity RIOK2 inhibitor, binds RIOK2 directly and induces a ribosomal stress signature, resulting in growth inhibition of ERG-positive VCaP tumor xenografts (Mohamed et al., 2018). In addition, RIOK2 is associated with CD4^+^ T cell activation (Subbannayya et al., 2020), suggesting that it may play a fundamental role in the regulation of the immune system. However, only a few malignancies have been linked to RIOK2 expression. Therefore, further studies on RIOK2 and its roles in the occurrence, development, and immune microenvironment of tumors are essential to identify therapeutic cancer targets.

To identify the involvement of RIOK2 in cancer systematically, we integrated multi-omics data of 33 cancer types to conduct the first comprehensive association analyses of RIOK2. We identified that RIOK2 was highly expressed in pan-cancer and was significantly correlated with tumor cell proliferation, migration, invasion, and immune cell infiltration, all of which can prove fatal to patients. We also found that alteration of RIOK2 *via* DNA methylation and phosphorylation were related to tumorigenesis. By describing the function of RIOK2 in pan-cancer, we demonstrate that RIOK2 represents a valuable target for treating malignant tumors.

## Materials and methods

### Data resources

The genomic, proteomic, and clinicopathological information of 33 cancer types were extracted from databases obtained from The Cancer Genome Atlas (TCGA) (https://portal.gdc.cancer.gov), International Cancer Genome Consortium, Cancer Cell Line Encyclopedia, and Clinical Proteomics Tumor Analysis Consortium (CPTAC) (https://proteomics.cancer.gov/programs/cptac). Corresponding information of healthy tissue samples were obtained from the Genotype–Tissue Expression (GTEx) database (https://gtexportal.org/home). The baseline information for all eligible datasets is summarized in Supplementary Table S1. In instances where healthy tissue samples corresponding to certain tumors in the TCGA database were lacking, we used the UCSC Xena platform to bridge the gap between TCGA and GTEx data by recomputing all the raw gene expression data based on a standard pipeline (Tang et al., 2019; Navarro Gonzalez et al., 2021).

### Differential expression analysis

We obtained RIOK2 expression data of 11,826 samples comprising 33 cancer types from the TCGA database and matched normal pairs between seven cancer types from the GTEx database and 24 from the TCGA database; these 31 cancer types were selected for further analysis. The TIMER2.0 (Li et al., 2020) (http://timer.cistrome.org) Gene DE module and the Gene Expression Profiling Interactive Analysis 2 (gepia2. cancer-pku.cn/) differential expression analysis module was employed to analyze the difference in RIOK2 expression between the tumor and healthy groups. (Wilcoxon test: |fold change| > 2, *p* < 0.05, q < 0.01). Furthermore, we conducted auxiliary verification using area under the receiver operating characteristic curve analysis and other expression profiles from the International Cancer Genome Consortium database.

Furthermore, to investigate RIOK2 protein levels in pan-cancers, we obtained the RIOK2 proteomic expression data of seven cancer types and normal pairs from the CPTAC database. The University of Alabama at Birmingham CANcer data analysis portal (UALCAN) (ualcan.path.uab.edu) (Chandrashekar et al., 2017) was used for differential expression analysis in RIOK2 between the tumor and healthy groups. (Wilcoxon test, *p* < 0.05). In addition, immunohistochemical data obtained from the Human Protein Atlas (https://proteinatlas.org) website were used as an indispensable complement to RIOK2 proteomic expression (Uhlen et al., 2017). The Benjamini–Hochberg test was used to control for false discovery rate across multiple tests.

### Cancer progression analysis

To determine the relationship between RIOK2 expression and cancer progression parameters such as lymph node metastasis and tumor stages, we applied differential signature score analysis. Data were generated on the Gene Expression Profiling Interactive Analysis 2 and UALCAN websites [Pr (>F) < 0.05] using the mean value of log2 (TPM +1) or TPM (transcripts per million) at the transcriptional level and Z-value at the protein level of each gene in tumors and healthy groups. Lymph node metastasis was divided into four levels: N0 (no regional lymph node metastasis), N1 (metastases in 1–3 axillary lymph nodes), N2 (metastases in 4–9 axillary lymph nodes), and N3 (metastases in ≥10 axillary lymph nodes). There were also four pathological groups (stage I–IV) according to tumor–node–metastasis staging. In addition to our analysis at the tissue level, we used the Wilcox test to compare the expression of RIOK2 at the cellular level in primary and metastatic cell lines; data were obtained from the Cancer Cell Line Encyclopedia database.

### Survival prognosis analysis

To study the time-dependent prognostic value of RIOK2 expression in pan-cancers, we obtained RIOK2 expression data from 11,882 samples comprising 21 cancer types and normal pairs from the TCGA database. To assess survival, we used Cox proportional hazards regression using the survival package for R software. Both the survival and survminer R packages were utilized to calculate log-rank *p*-values, hazard ratios, and 95% confidence intervals. All possible cutoff values between the lower and upper quartiles were computed, and the best-performing threshold was used as the cutoff. Survival outcomes included overall survival and relapse-free survival, which were visualized using the Kaplan–Meier Plotter (https://kmplot.com) (Nagy et al., 2021). Statistical significance was defined as a log-rank *p*-value of <0.05. All data processing was performed using R version 4.1.2.

### Genetic alterations analysis

We obtained RIOK2 alteration data from 162 samples comprising 20 cancer types from the TCGA database. cBioPortal (cbioportal.org) was used to summarize alterations, especially mutations, at various sites in the primary structure of RIOK2. Kinase activity was likely to change if member residues in the functional domain were more frequently mutated. Mutations were mapped to the sequences and structures obtained from the STRING version 11.5 protein database (https://string-db.org) (Szklarczyk et al., 2021) using BIOVIA Discovery Studio 2019 Client software. Furthermore, we applied Cox proportional hazards regression analysis to investigate the time-dependent prognostic value of RIOK2 mutations.

### Phosphorylation and methylation modification analysis

To analyze the differential expression of phosphorylated RIOK2 in pan-cancer, we obtained phosphorylated RIOK2 expression data from 767 samples comprising five cancer types and normal pairs from the CPTAC database. Differential gene expression analysis was performed using the UALCAN database. The differential signature score was tested according to Z-value, and its significance was calculated by Student’s t-test (Chandrashekar et al., 2017) (*p* < 0.05). Simultaneously, the RIOK2 phosphorylation of tumor sites in the tumor, healthy, and prediction groups were obtained from the databases of Quantification of Post-Translational Modifications (http://qptm.omicsbio.info), Phosida (http://141.61.102.18/phosida/index.aspx), Phosphosite Plus (https://phosphosite.org/homeAction.action), and PhosphoNET (http://www.phosphonet.ca) (Gnad et al., 2007; Gnad et al., 2011; Yu et al., 2019).

To compare the difference in RIOK2 methylation degree between healthy and tumor samples and to assess their relevance to expression, we obtained 11 methylation probes from hg38 coordinates provided by Zhou et al. (2017) (http://zwdzwd.github.io/InfiniumAnnotation). We comprehensively analyzed RIOK2 DNA methylation alongside other omics data from TCGA with the Wilcoxon rank sum test and by using the Shiny Methylation Analysis Resource Tool (Letunic et al., 2021) (http://bioinfo-zs.com/smartapp) and MEXPRESS web tool (Koch et al., 2019) (https://mexpress.be). Beta values ranged from 0 to 1, with 0 being unmethylated and 1 being fully methylated. Statistical significance was defined as a *p* < 0.05, as calculated *via* the Wilcoxon rank sum test and adjusted using the Benjamini–Hochberg method. To investigate the relationship between the methylation of RIOK2 within the range of TSS1500 and overall survival of patients, Cox proportional hazards regression was applied. All possible cutoff values (mean, median, interquartile range, and maximum) were computed, and the best-performing threshold was used as a cutoff. The difference in overall survival between the lower and higher methylated groups of patients was visualized using Kaplan–Meier plots on MethSurv (Modhukur et al., 2018) (https://biit.cs.ut.ee/methsurv). A log-rank *p*-value < 0.05 was considered to be statistically significant.

### Immune infiltration analysis

In order to investigate the correlation between RIOK2 expression and immune cell infiltration (EPIC and xCell algorithms), we obtained RIOK2 expression and immune infiltrate data of 12,159 samples comprising 33 cancer types from the TCGA database (Li et al., 2016; Li et al., 2017; Sturm et al., 2019; Li et al., 2020). In addition, we used Spearman correlations to investigate the relation between the abundance of 28 tumor-infiltrating lymphocytes, three kinds of immunomodulators (immunoinhibitors, immunostimulators, and major histocompatibility complex molecules) and the expression of RIOK2 using the Tumor and Immune System Interaction Database (Ru et al., 2019). Statistical significance was set at *p* < 0.05.

### RIOK2-related gene enrichment analysis

The RIOK2 pathway was investigated at the genetic and protein levels. Seventy-six RIOK2-interacting proteins were selected from the STRING database according to an interaction score of >0.4 and verified by experiment. The protein—protein interaction (PPI) network was clustered using molecular complex detection (with a plugin in Cytoscape version 1.5.1), and five interacting proteins were identified. Spearman’s rank correlation test was performed to analyze the correlation between the expression of RIOK2 and that of the above five proteins. Furthermore, the Similar Gene Detection module of GEPIA2 was used to detect the co-expression of genes with RIOK2. According to their Spearman correlation coefficient ranking, the top 500 RIOK2-related target genes were verified a second time *via* the Spearman’s rank correlation test (Tang et al., 2019) (correlation >0.33, *p* < 0.05). Finally, we used the clusterprofiler R package (Yu et al., 2012), KEGG (Kyoto Encyclopedia of Genes and Genomes), and GO (Gene Ontology) enrichment analysis to study which biological processes RIOK2 affected. Statistical significance was set at *p* < 0.05.

## Results

### Elevated RIOK2 expression across multiple cancer types

**FIGURE 1:**
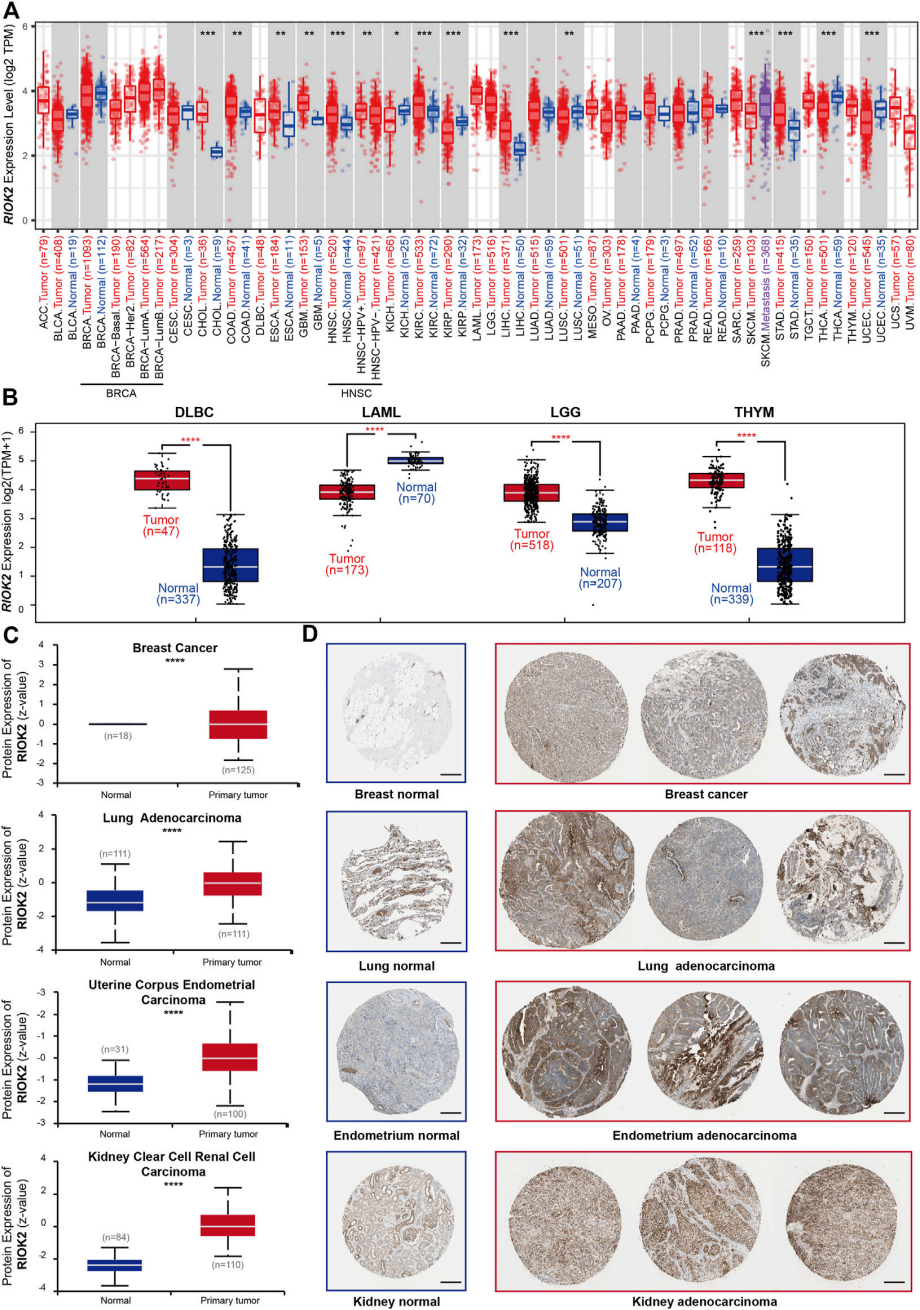
Expression level of RIOK2 gene in different tumors and normal tissues. The distribution of RIOK2 expression levels is shown using box plots. We identified RIOK2 mRNA **(A,B)** and protein **(C)** that were upregulated or downregulated in tumors (red) compared to healthy tissues (blue) for each cancer type, as displayed in gray columns where control data were available. **(D)** Representative images of immunohistochemistry staining for RIOK2 protein constructed from four cancer types. Scale bars: 200 μm. Statistical significance, as computed using the Wilcoxon test, is annotated by the number of stars (*p < 0.05, **p < 0.01, ***p < 0.001, ****p < 0.0001).

RIOK2 was previously confirmed to be highly expressed and over-activated in non-small cell lung cancer, prostate cancer, and glioblastoma, driving the occurrence and development of related tumors (Read et al., 2013; Liu et al., 2016; Liu et al., 2018; Mohamed et al., 2018; Delman et al., 2019; Song et al., 2020). We obtained RIOK2 expression data of 31 cancer types, matched normal pairs from the TCGA and GTEx databases, and performed an in-depth analysis of RIOK2 to determine whether its expression varied between tumor and healthy groups. RIOK2 mRNA levels were elevated in most tumor types, with the highest levels detected in cholangiocarcinoma (CHOL), diffuse large B-cell lymphoma (DLBC), thymoma (THYM), glioblastoma multiforme (GBM), brain lower-grade glioma (LGG), and pancreatic adenocarcinoma (PAAD), followed by colon adenocarcinoma (COAD), liver hepatocellular carcinoma (LIHC), kidney renal clear cell carcinoma (KIRC), head and neck squamous cell carcinoma (HNSC), stomach adenocarcinoma (STAD), and esophageal carcinoma (ESCA). Interestingly, RIOK2 expression in malignant tissues was lower than that in healthy tissues, as was the case for lung squamous cell carcinoma (LUSC), kidney renal papillary cell carcinoma (KIRP), kidney chromophobe (KICH), uterine corpus endometrial carcinoma (UCEC), and thyroid carcinoma (THCA) (Figures 1A,B, Wilcoxon *p*-value < 0.05). To further verify the above results, area under the receiver operating characteristic curve analysis was performed on the normalized log2-transformed expression values of RIOK2. The results indicated that elevated RIOK2 expression was common in most cancer patients, with our values ranging from 0.699 to 0.975 (Supplementary Figure S1B). In addition, tumor samples from the International Cancer Genome Consortium database delivered similar results (Supplementary Figure S2A).

**FIGURE 2:**
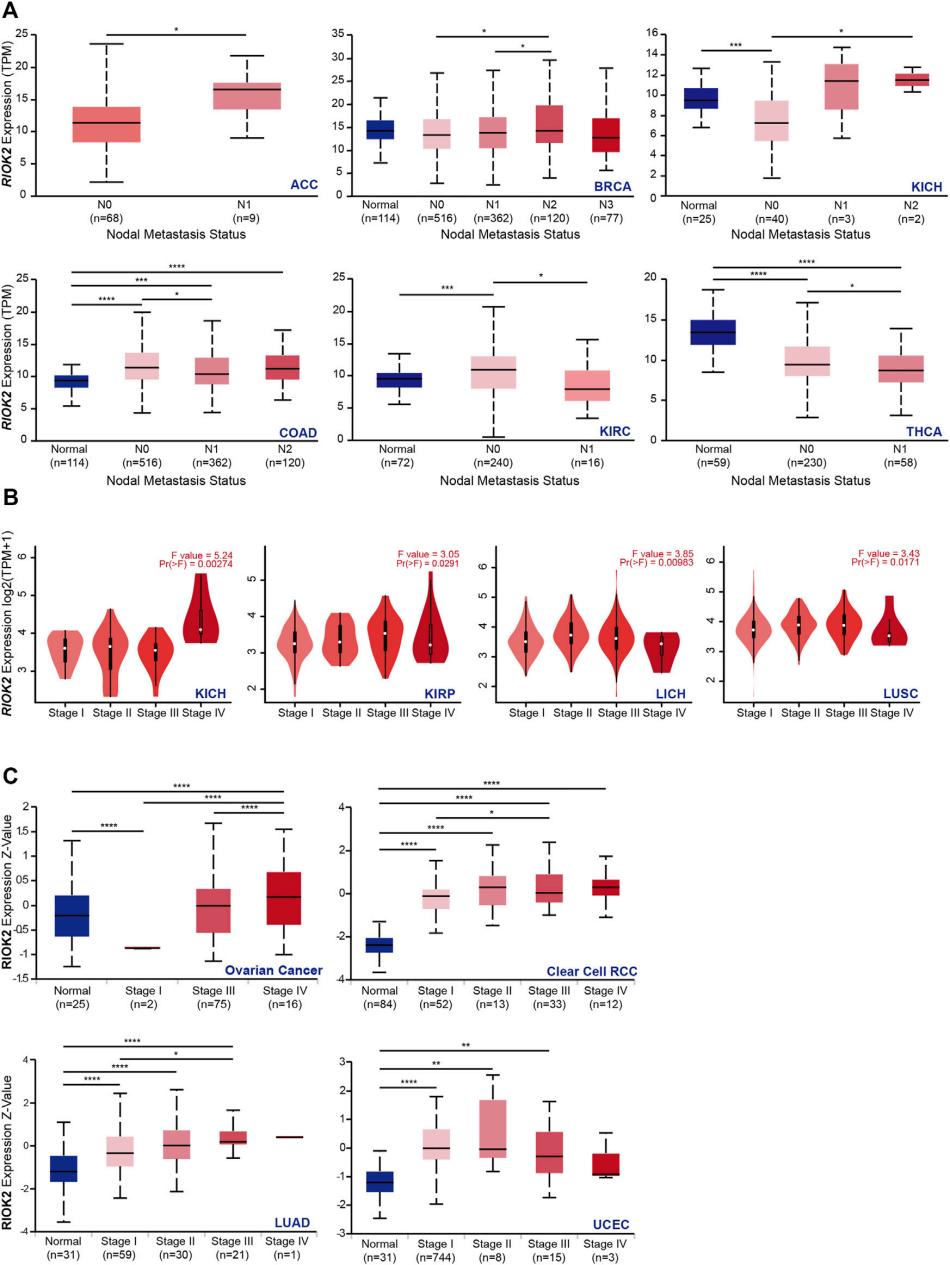
Correlation between RIOK2 gene expression and lymph node metastasis and four pathological stages of cancers. **(A)** Box plots representing the RIOK2 mRNA level calculated by TPM (y-axis) for each nodal metastasis status of TCGA cancer types (x-axis). The blue box represents the healthy group, and the red one represents the tumor group. The nodal metastasis status is divided into N0, N1, N2, and N3. Among them, N0 indicates no regional lymph node metastasis; N1 indicates metastases in 1–3 axillary lymph nodes; N2 indicates metastases in 4–9 axillary lymph nodes; N3 indicates metastases in ten or more axillary lymph nodes. (*p < 0.05; **p < 0.01; ***p < 0.001; ****p < 0.0001). **(B)** The expression of RIOK2 in TCGA RNA-Seq datasets in four pathological stages (stages I–IV) were calculated by mean value of log2 (TPM +1) using violin plots. **(C)** The expression of RIOK2 at protein level in pathological stages I–IV was evaluated by Z-value using box plots. (*p < 0.05, **p < 0.01, ***p < 0.001).

To investigate the protein expression levels of RIOK2 in pan-cancer, we obtained proteomic expression data for seven cancer types and normal pairs from the CPTAC database. RIOK2 protein levels were significantly elevated in breast cancer, lung adenocarcinoma (LUAD), UCEC, and KIRC, which was in concordance with the high levels of RIOK2 mRNA in KIRC and LUAD (Figure 2C, Student’s t-test, *p* < 0.05). To determine the prevalence of human cancers that were RIOK2 protein-positive, immunohistochemistry of tumor tissues with RIOK2 antibodies was obtained from the Human Protein Atlas website. High regulation of RIOK2 expression occurred more frequently in breast cancer, LUAD, UCEC, and KIRC than in the corresponding healthy tissues, which was consistent with the above results (Figures 2C,D).

Considering that a variety of cell types are present in tumors, we applied partial Spearman’s correlation to determine whether RIOK2 was expressed by cancer cells. According to the regression curves (Supplementary Figure S1A), RIOK2 mRNA was enriched in adrenocortical carcinoma, brain LGG, and skin cutaneous melanoma (SKCM) with an increasing level of tumor purity (Figure 1C), indicating that elevated or decreased RIOK2 expression in most tumors partly originated from tumor cells and partly from non-tumor cells. These results also imply that RIOK2 may play a role in the tumor microenvironment.

### RIOK2 level was related to tumor metastasis and determined cancer prognosis

In previous investigations, RIOK2 mRNA expression in metastases was higher in SKCM compared to the primary focus, which may indicate that RIOK2 expression is related to cancer metastasis (Figure 1A, Wilcoxon *p*-value < 0.05). Given the high expression of RIOK2 in pan-cancer, we investigated whether RIOK2 was required for tumor progression to worsen prognosis and shorten patient survival. First, we performed differential gene expression analysis to assess the relationship between RIOK2 expression, nodal metastasis status, and pathological stages in pan-cancer. Interestingly, positive correlations between RIOK2 mRNA and nodal metastatic status were established in six different cancer types, namely adrenocortical carcinoma, breast invasive carcinoma (BRCA), KICH, COAD, KIRC, and THCA (Figure 2A, *p* < 0.05). In addition, in the case of KICH, KIRP, LICH, LUSC, ovarian serous cystadenocarcinoma (OV), clear cell renal cell carcinoma (ccRCC), LUAD, and UCEC, RIOK2 mRNA expression was lower in healthy tissue and increased with pathological stage, with the highest levels observed in invasive cancer samples (Figure 2B, *p* < 0.05). We arrived at a similar conclusion regarding RIOK2 protein levels in OV, ccRCC, LUAD, and UCEC (Figure 2C, *p* < 0.05). Consistent with the above results, we also found a positive correlation between the expression of RIOK2 and metastasis in BRCA, COAD, rectum adenocarcinoma (READ), and DLBC tumor cell lines, but a negative correlation between these two factors in OV (Supplementary Figures S2B–E, *p* < 0.05).

**FIGURE 3:**
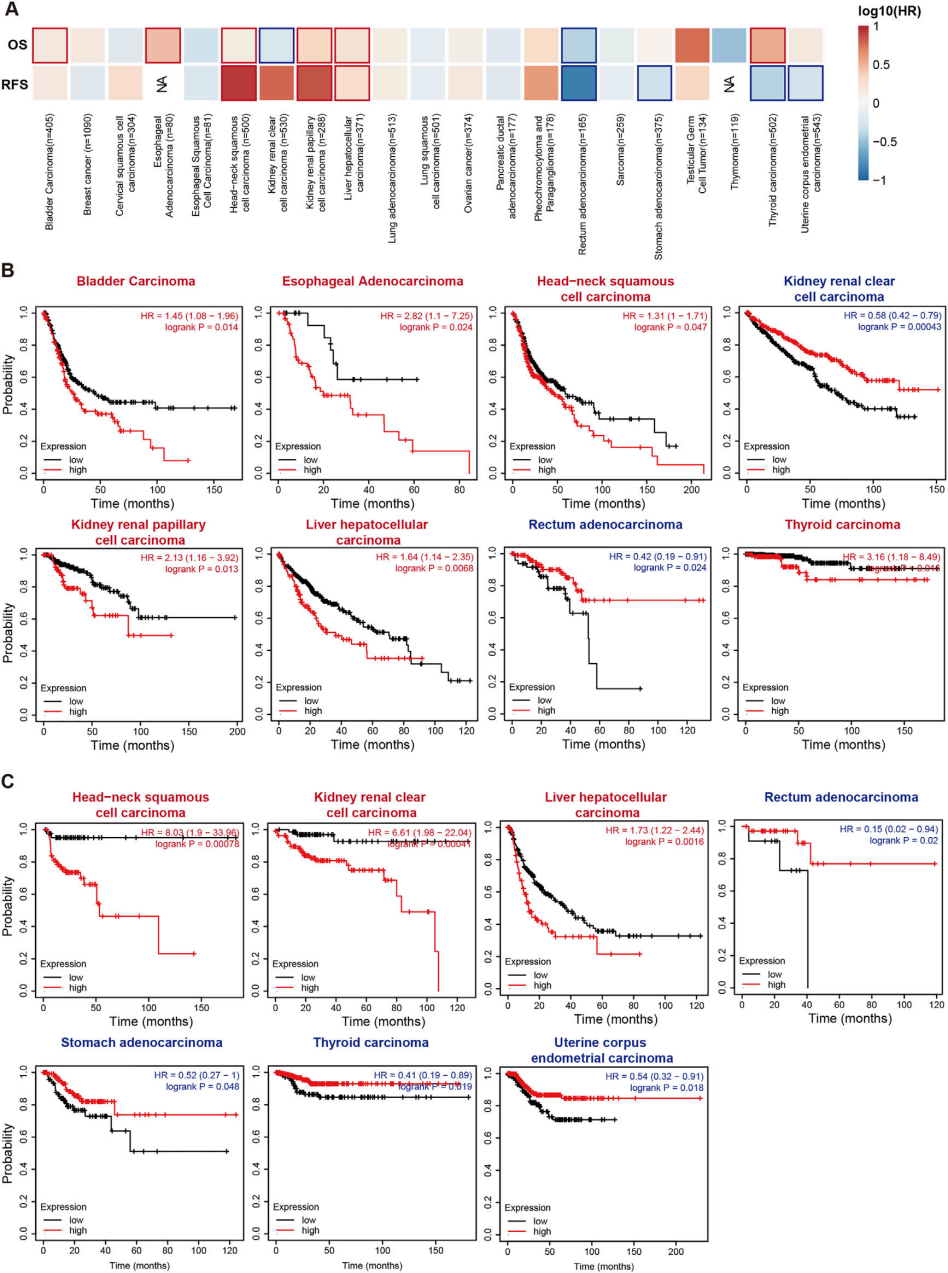
Relevance between RIOK2 gene expression and survival prognosis of cancers in TCGA. **(A)** The correlation between RIOK2 expression and overall survival and relapse-free survival across multiple cancer types is shown in a heatmap. The bold border in the heatmap represents significance (*p* < 0.05). **(B,C)** The overall and relapse-free survival Kaplan-Meier curves of the various cancer types that have significant survival risk (*p* < 0.05) are displayed, with high RIOK2 expression curves colored in red and low ones in blue.

We performed Cox proportional hazards regression to further confirm the impact of RIOK2 target gene expression on patient survival across the pan-cancer cohort. Patients with low RIOK2 expression had the best outcome, followed by patients with high RIOK2 expression in more cancers (Figure 3; Supplementary Figure S3. Overexpression of RIOK2 in bladder urothelial carcinoma (BLCA), ESCA, HNSC, KIRP, LIHC, and THCA was strongly associated with poor patient outcomes. Poor prognosis was also linked to low RIOK2 expression in tumors with increased RIOK2, including KIRC, REA, STAD, THCA, and UCEC. (Figure 3; Supplementary Figure S3).

These results suggest that a high expression of RIOK2 is associated with tumor progression and poor patient survival, indicating that it may be a potential biomarker to characterize prognosis.

### Impact of DNA methylation of the RIOK2 gene in pan-cancer

**FIGURE 4:**
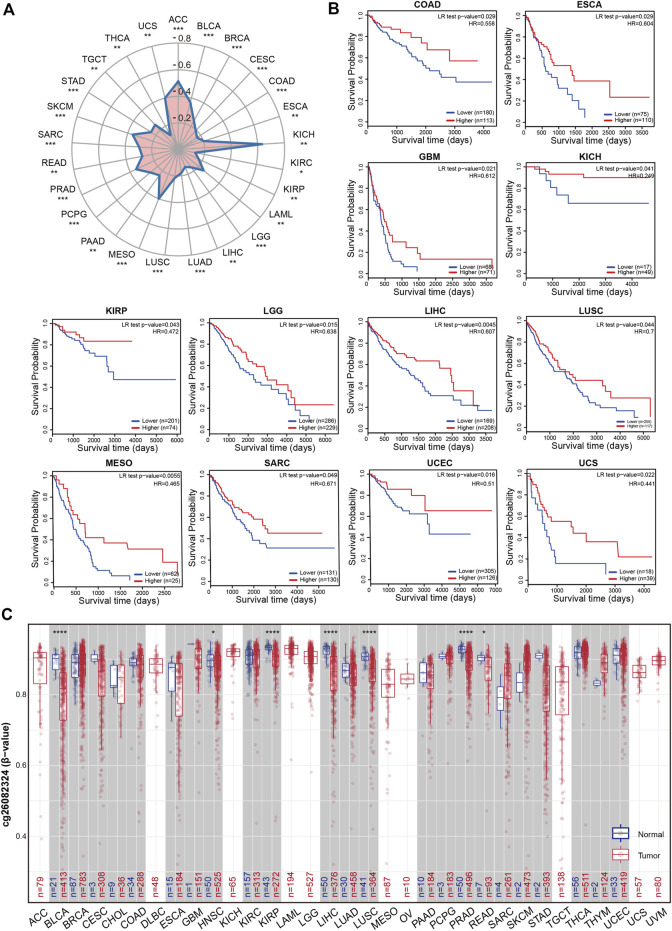
Methylation feature of *RIOK2* in different tumors of TCGA.**(A)** Correlation between the RIOK2 methylation levels and expression is displayed using a radar chart across the various cancer types. The pattern is observed with the CpG island probe cg06664872. The statistical significance computed by the Spearman test is annotated by the number of stars (*p < 0.05, **p < 0.01, ***p < 0.001, ****p < 0.0001). **(B)** Correlation between the RIOK2 methylation levels and overall survival is shown by the Kaplan-Meier curves across the various cancer types which have significant survival risk (*p* < 0.05). **(C)** Differences in RIOK2 methylation levels between tumors and healthy groups are displayed using box plots that are upregulated or downregulated in tumors (red) compared to healthy tissues (gray) for each cancer type. The pattern is observed with the N-shelf probe cg26082324. Beta-values are between 0 and 1, with 0 being unmethylated and 1 being fully methylated. The statistical significance computed by the Wilcoxon test is annotated by the number of stars (*p < 0.05, **p < 0.01, ***p < 0.001, ****p < 0.0001).

DNA methylation in the kinase promoter region has often been described as a “silent” epigenetic marker, because it can change genetic performance without changing the associated DNA sequence. Based on the widespread elevation of RIOK2 expression in tumors, we postulated that RIOK2 expression changes due to its methylation. Reduced RIOK2 expression in some tumor samples with increased promoter methylation was confirmed by statistical analysis of the correlation between DNA methylation and gene expression (Figure 4A, Wilcoxon *p*-value < 0.05). To analyze the impact of DNA methylation at the RIOK2 promoter region on patient survival across the pan-cancer cohort, we performed Cox proportional hazards regression for the promoter region probe cg06664872. As illustrated in Figure 4B, the degree of methylation at the RIOK2 promoter region was significantly and negatively correlated with overall survival in most cancer patients, indicating that the hypermethylation of RIOK2 in the TSS1500 region may inhibit the occurrence and development of tumors by inhibiting the expression of RIOK2.

In addition to the effect of DNA methylation on gene expression, tumors are characterized by methylation imbalance. In general, DNA methylation within the CpG island region of a gene’s promoter region is correlated with gene expression, while DNA methylation within the gene body is also associated with chromosomal integrity (Jones, 2012). To examine the methylation status of RIOK2, 11 probes from the SMART database were selected and analyzed across pan-cancers. Differentially methylated probe sets were significantly enriched in N-shelves but were not mentioned in previous studies. The N-shelf probe cg26082324 showed a pattern with reduced methylation in almost all examined malignancies relative to healthy tissues, suggesting that differential RIOK2 N-shelf site methylation may be related to carcinogenesis in pan-cancer (Figure 4C). Despite the aforementioned differences in the RIOK2 methylation region, the methylation levels of RIOK2 were correlated with tumorigenesis and progression.

### Genetic alteration among individual cancer types

**FIGURE 5:**
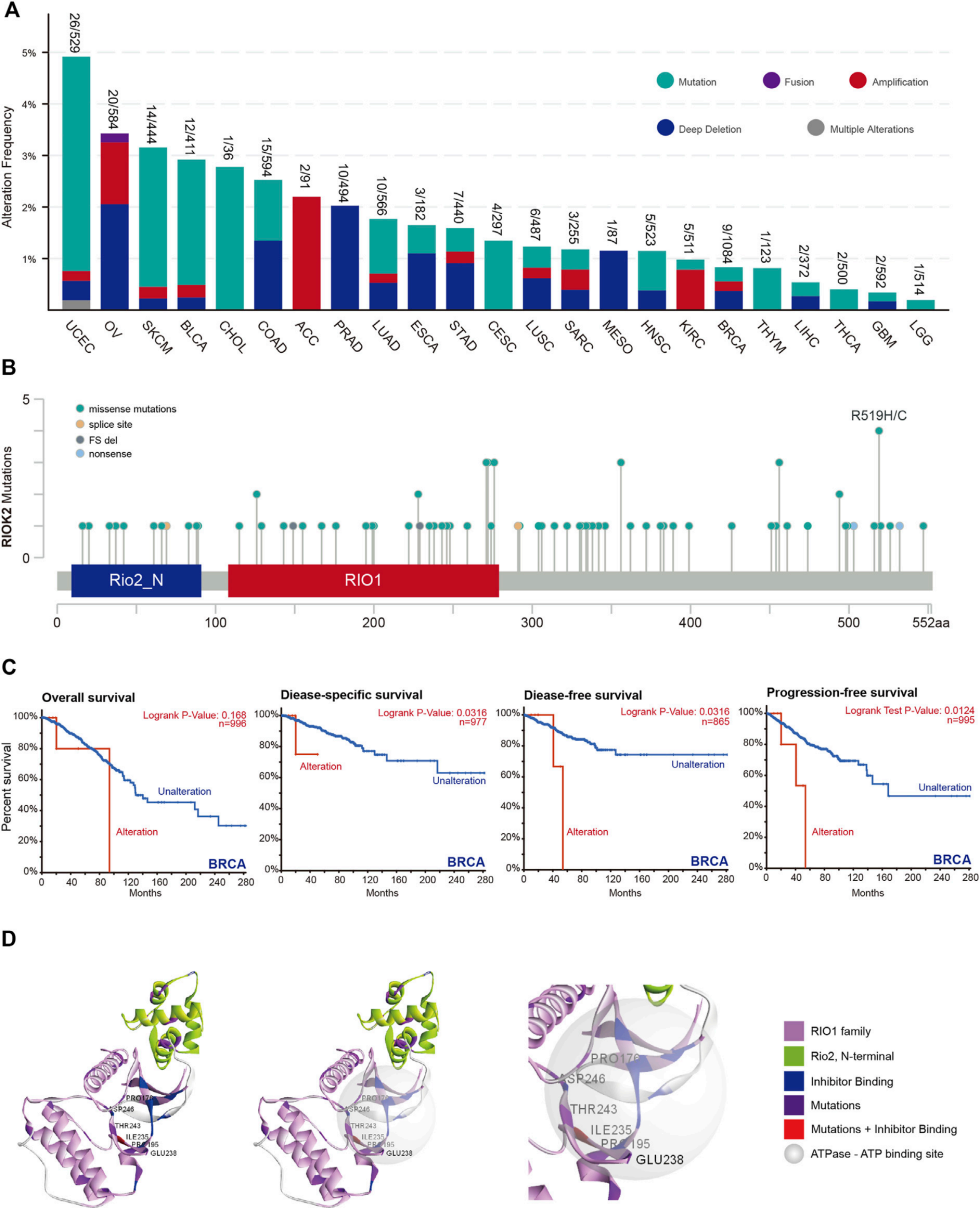
Mutation feature of RIOK2 in different tumors of TCGA. **(A)** The histogram compares the frequency of RIOK2 gene alteration type between different tumors. **(B)** Mutation sites of RIOK2 in 32 studies of TCGA Pan-Cancer Atlas. Mutation diagram circles are colored with respect to the corresponding mutation types. In case of different mutation types at a single position, color of the circle is determined with respect to the most frequent mutation type. **(C)** The disease-specific, disease-free, and progression-free survival curves of the BRCA which have significant survival risk (*p* < 0.05). **(D)** The image indicates the relationship between the domain of the RIO1 family, N-terminal of RIO2, ATP-binding site, inhibitor binding site, and mutation site, using a 3D structure from different angles.

Alterations in kinase genes associated with human cancers have previously been identified, the majority of which are located within the kinase domains (Negrini et al., 2010). To identify the types and regions of RIOK2 mutations, comprehensive characterization of RIOK2 alterations in a total of 10,967 samples comprising 23 cancer types were evaluated using cBioPortal. RIOK2 alterations occurred most frequently in UCEC (4.9%), followed by OV (3.4%), and SKCM (3.2%) (Figure 5A). There were five types of RIOK2 alterations: mutation, fusion, amplification, deep deletion, and multiple alterations. Mutations and deep deletions were more common in the pan-cancer cells. Cancer types with high RIOK2 mutation levels included UCEC, SKCM, BLCA, and CHOL (Figure 5A). Since the amino acid residues of human RIOK2 that undergo mutation were previously unknown, we summarized our results on cBioPortal. There were significantly more mutation events at amino acid residues Arg519, Ser456, Arg356, Arg276, Asp271, Asp228, and Arg126 (Figure 5B). Hotspots in RIOK2 at Arg126, Asp228, Phe271, and Arg276 (four conserved sites in RIO1) were self-stabilizing (Figure 5B). These mutations could have widespread effects because RIO1 is a binding partner of ATP. Furthermore, we observed that Pro176, Pro195, Ile235, Glu238, Thr243, and Asp246 were located within the active region of RIOK2 (Figure 5D). Recently, Wang et al. (2019) developed a crystal structure of a specific inhibitor that binds to the active center of RIOK2, called RIOK2i. Notably, Ile235, which was mutated to Val235, was not only within the active region of RIOK2 but also within the binding site of RIOK2i.

Finally, we probed for associations between *RIOK2* mutations and overall survival, disease-specific survival, disease-free survival, and progression-free survival for all cancers (Figure 5C). Patients with higher *RIOK2* alterations were significantly correlated with poorer prognosis in BRCA.

### Impact of RIOK2 protein phosphorylation in pan-cancer

**FIGURE 6:**
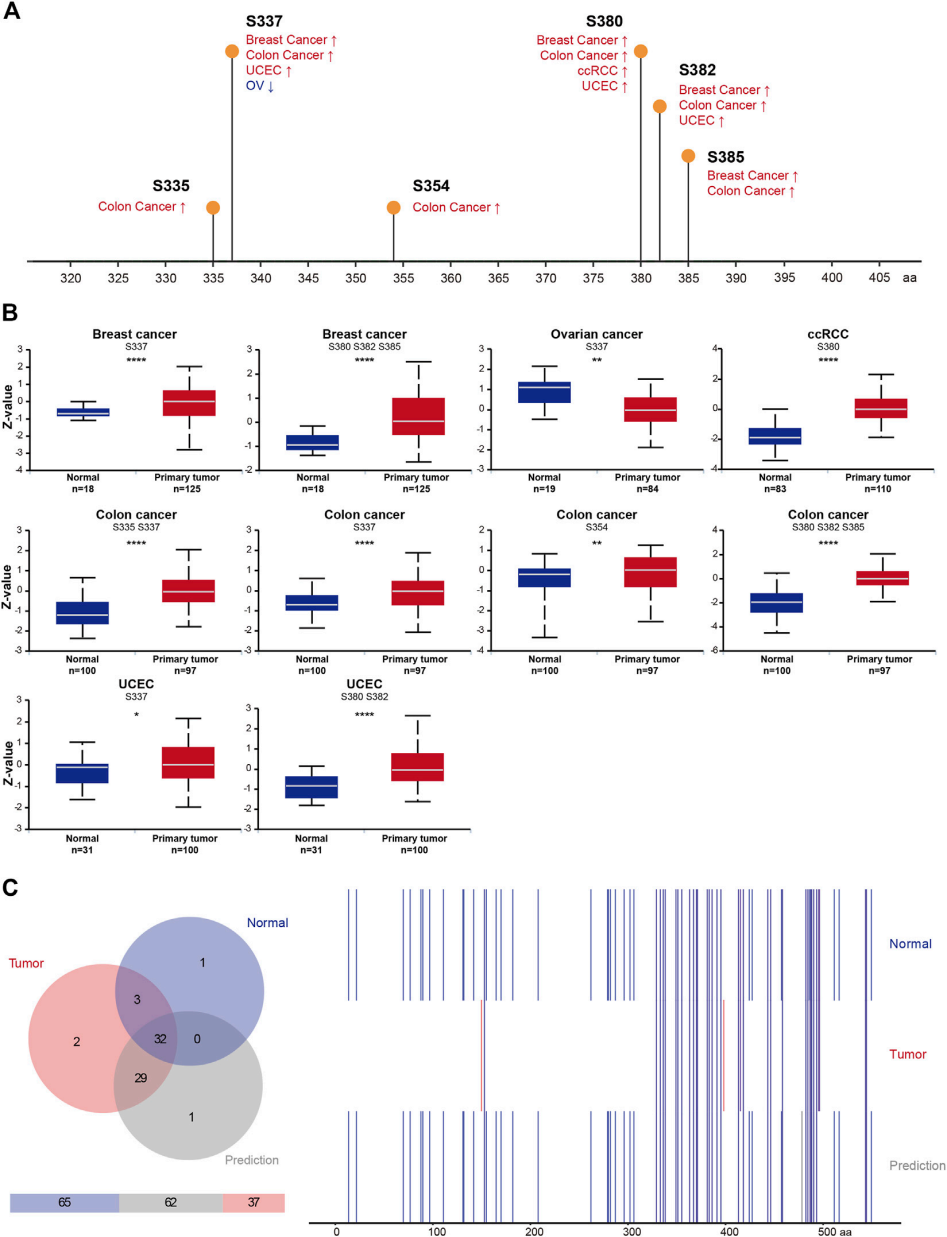
Phosphorylation feature of RIOK2 in different tumors of TCGA. **(A)** An overview of the changes in RIOK2 phosphorylation in breast cancer, colon cancer, ccRCC, UCEC, and OV from the CPTAC database. **(B)** Box plots representing the RIOK2 phosphorylation level calculated by Z-value (y-axis) for each TCGA cancer type and corresponding healthy groups (x-axis). The statistical significance computed by Student’s t-test is annotated by the number of stars (*p < 0.05, **p < 0.01, ***p < 0.001, ****p < 0.0001). **(C)** The Venn diagram on the left counts the number of RIOK2 phosphorylation sites in cancer and healthy tissues, including experimental data and predicted data. The right panel of the chart depicts the difference between tumor cells, normal cells, and predictive sites of RIOK2 phosphorylation. The information about RIOK2 phosphorylation sites in tumor cells was extracted from 72 samples of the Quantification of Post-Translational Modifications database, and the phosphorylation sites in healthy cells were obtained from the PhosphoNET, Phosphosite Plus, and Phosida datasets (these data have been verified by high-throughput proteomics technologies); the predicted phosphorylation sites come from thr PhosphoNET database.

Many cancers arise as a result of deregulation of kinase phosphorylation, which alters protein function. Thus, studies on RIOK2 phosphorylation may reveal the factors underlying carcinogenesis and progression. RIOK2 has the capability to auto-phosphorylate, which is important for its function (Zemp et al., 2009). We identified six sites where the phosphorylation frequency changed in colon cancer, breast cancer, UCEC, OV, and KIRC, namely Ser335, Ser337, Ser354, Ser380, Ser382, and Ser385. Except for OV, the phosphorylation levels at these six sites increased significantly (Figures 6A,B, Student’s t-test, *p* < 0.05). Furthermore, Ser337 and Ser380 were most frequently phosphorylated among the five types of cancer in which phosphorylation was enhanced. To determine whether RIOK2 phosphorylation is required for tumorigenesis, we summarized experimentally verified and predicted phosphorylation sites of tumors and normal tissues based on the four databases of Quantification of Post-Translational Modifications, Phosida, Phosphosite Plus, and PhosphoNET. Venn diagram analysis identified two phosphorylated amino acids that were significantly tumor-specific: Ser149 and Asn397 (Figure 6C). Therefore, over-phosphorylation of RIOK2 in tumors suggests its potential functional importance in pan-cancers.

### Relationship between mRNA expression of RIOK2 and the tumor-immune microenvironment

**FIGURE 7:**
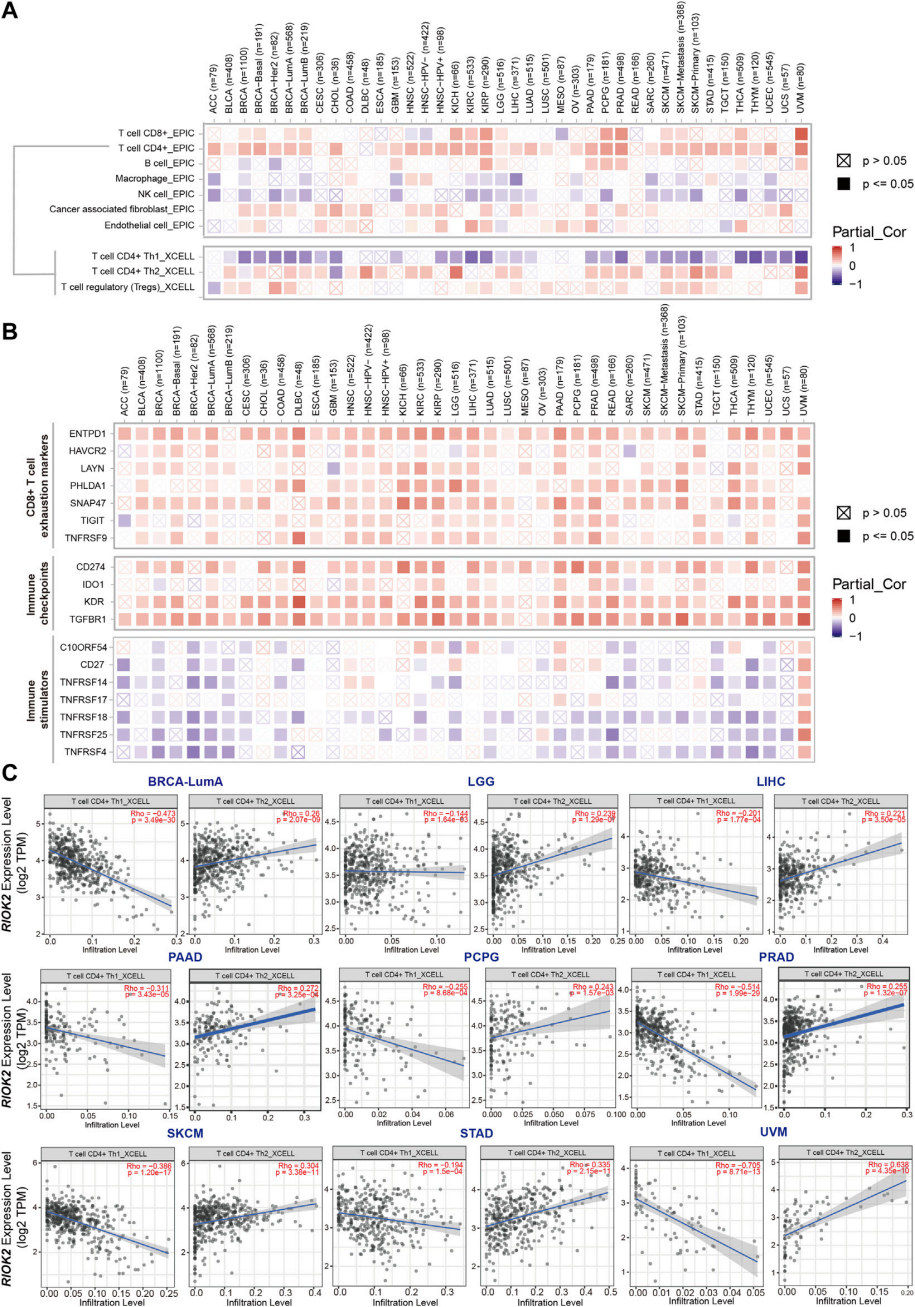
The expression of RIOK2 is correlated with immune cells infiltration and biomarkers in cancer. **(A)** A heat map showing the correlation between RIOK2 expression and immune infiltration levels in diverse cancer types. The correlation of infiltrating cells were obtained using the EPIC algorithm, and the correlation of CD4+T cell subtypes by using the xCell algorithm with Spearman purity adjustment. **(B)** A heat map conveys the correlation between RIOK2 expression and seven CD8+T exhaustion markers, four immune checkpoints, and seven immune stimulators in diverse cancer types. **(C)** Scatter plots showing the relationship between CD4+Th1 and CD4+Th2 infiltrate values and RIOK2 expression. Spearman’s correlation was employed to conduct a purity adjustment analysis.

The above results suggest that aberrance in RIOK2 expression and function in cancer cells probably contributes to the occurrence and progression of cancer. It is now clear that tumorigenesis and metastasis of tumors lie in the two-way interaction between cancer cells and their environment, forming a tumor microenvironment (Oehler et al., 2009). To investigate the association between RIOK2 expression and immune cell infiltration levels in pan-cancer, TCGA data were analyzed *via* purity-adjusted Spearman’s rank correlation tests using EPIC and xCell algorithms (Zemp et al., 2009). The results demonstrated that high expression of RIOK2 was associated with CD8+T cells, CD4+Th2 cells, CD4+Treg cells, and cancer-associated fibroblast infiltration in pan-cancer, whereas CD4+Th1 cells, macrophages, and natural killer cells showed the opposite association (Figure 7A). Multiple known CD8+T cell exhaustion markers, such as HAVCR2, ENTPD1, TIGIT, TNFRSF9, LAYN, PHLDA1, and SNAP47 (Zheng et al., 2017), were positively correlated with the expression of RIOK2, indicating that the elevated expression of RIOK2 resulted in effector T cells with a reduced capacity to secrete cytokines and an increased expression of inhibitory receptors (Figure 7B). The CD4+Th1/CD4+Th2 balance plays an important role in the tumor microenvironment (Ruterbusch et al., 2020), and our results showed that high expression of RIOK2 was negatively correlated with CD4+Th1/CD4+Th2 cells, indicating that RIOK2 is involved in Th1/Th2 regulation (Figure 7C).

Furthermore, we identified other types of tumor-infiltrating lymphocytes and biomarkers correlated with RIOK2 in immunotherapy from the Tumor and Immune System Interaction Database (Ru et al., 2019). The landscape of the relationship between RIOK2 expression and tumor-infiltrating lymphocytes in pan-cancer is shown in Supplementary Figures S4, S5. Specifically, Spearman correlations showed that RIOK2 expression was negatively associated with the infiltration levels of B cells, eosinophils, mast cells, monocytes, and neutrophils. Increasing evidence suggests that immune checkpoint blockade is among the most promising therapies for cancer. Our results revealed that RIOK2 is involved in the regulation of immune checkpoint expression in pan-cancer. The detailed relationship between RIOK2 and each representative immune checkpoint is shown in Figure 7B. Notably, RIOK2 demonstrated mutual exclusivity with several immune stimulators, namely C10ORF54, CD27, and the tumor necrosis factor receptor superfamily, but a statistically significant co-occurrence with some immunological checkpoints, namely CD274, IDO1, KDR, and transforming growth factor beta receptor I. In addition, a reduction in major histocompatibility complex I molecule expression in human tumors is often detected by pathologists (Burr et al., 2019). Understanding how a given tumor can evade detection by CD8^+^ T cells could help determine immunotherapies that are most likely to succeed against that tumor. As shown in Supplementary Figure S4E, the expression of major histocompatibility complex I molecules, such as HLA-A, HLA-B, and HLA-C, was found to be negatively associated. These findings indicate that RIOK2 is closely related to the infiltration of immune cells, which may be involved in regulating the tumor microenvironment in pan-cancers.

### Signaling pathways involving RIOK2-interacting proteins

**FIGURE 8:**
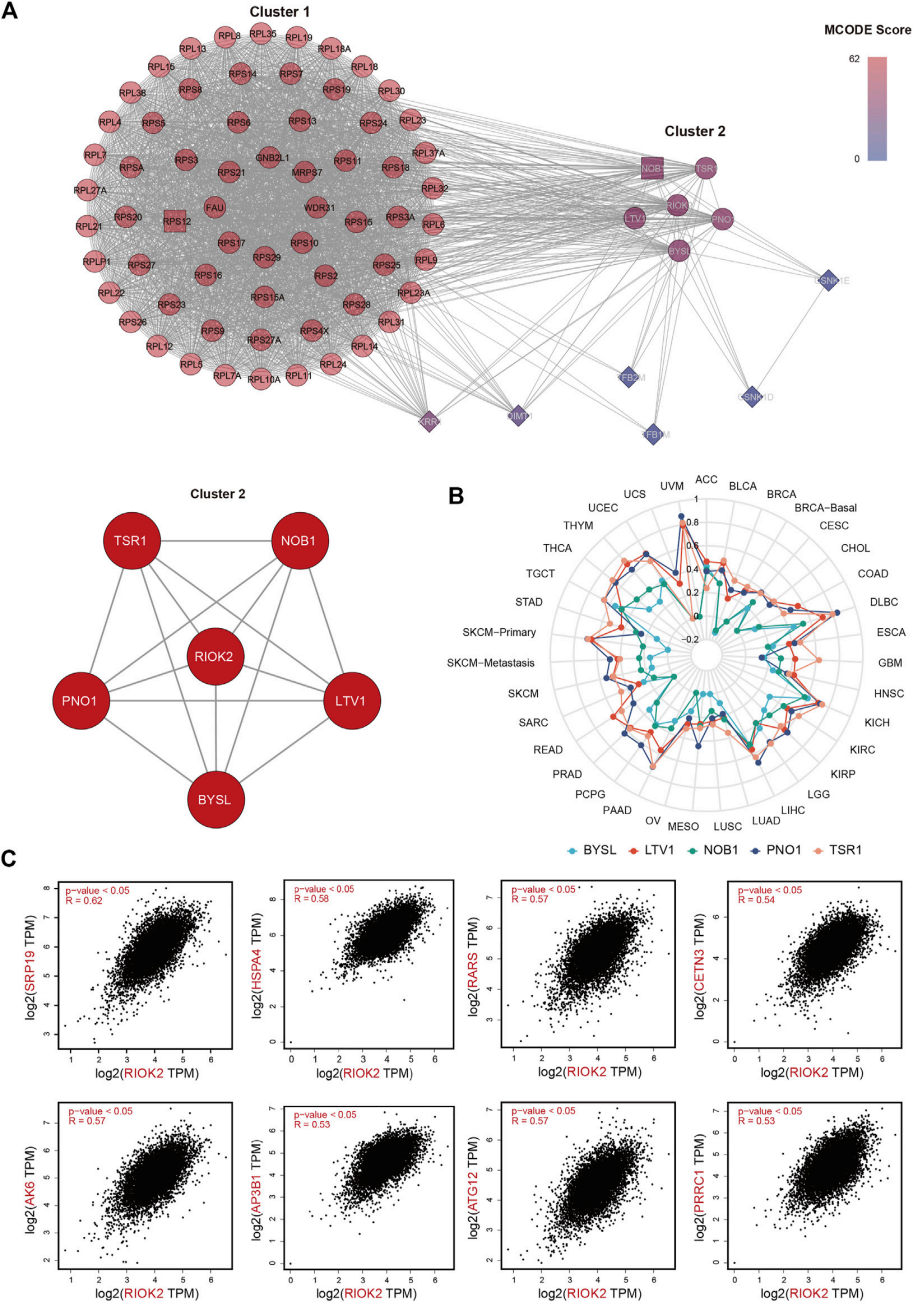
RIOK2-related genes and proteins analysis in tumor tissues. **(A)** A connectivity map of the PPI network involving 76 experimentally available RIOK2 interaction proteins from the STRING database linked *via* 2,290 interactions. Major hubs are highlighted in red. The minimum required interaction score was for medium confidence (0.400). The resulting PPI network was visualized using Cytoscape and further analyzed using molecular complex detection. **(B)** A radar chart shows that the correlation between RIOK2 expression and five proteins was assessed by purity-adjusted partial Spearman’s rho-value in pan-cancer. **(C)** Genes that correlated with RIOK2 expression with the top 500 Spearman correlation coefficients were obtained from TCGA. The expression correlation of the top eight RIOK2-related genes and RIOK2 was visualized by scatter plots using Spearman’s correlation for supplementary verification.

With mixed reports of its behavior, we sought to understand the potential mechanism of RIOK2 activity in pan-cancer using functional gene and protein interaction networks. We used gene expression profiles to construct RIOK2 PPI networks based on the STRING database and assessed their correlations with RIOK2 in a pan-cancer analysis. The resulting PPI network contained 76 nodes and 2,290 non-redundant edges visualized using Cytoscape and further analyzed by molecular complex detection. We later re-clustered the above proteins with molecular complex detection in the same software to obtain Cluster1 and Cluster2. Cluster2 was more densely connected to RIOK2 than Cluster1 (Figure 8A). The expression of five proteins in Cluster2 that were related to RIOK2 was assessed using purity-adjusted partial Spearman’s rho-value as the degree of their correlation in pan-cancer, as illustrated in our radar chart (Figure 8B).

**FIGURE 9:**
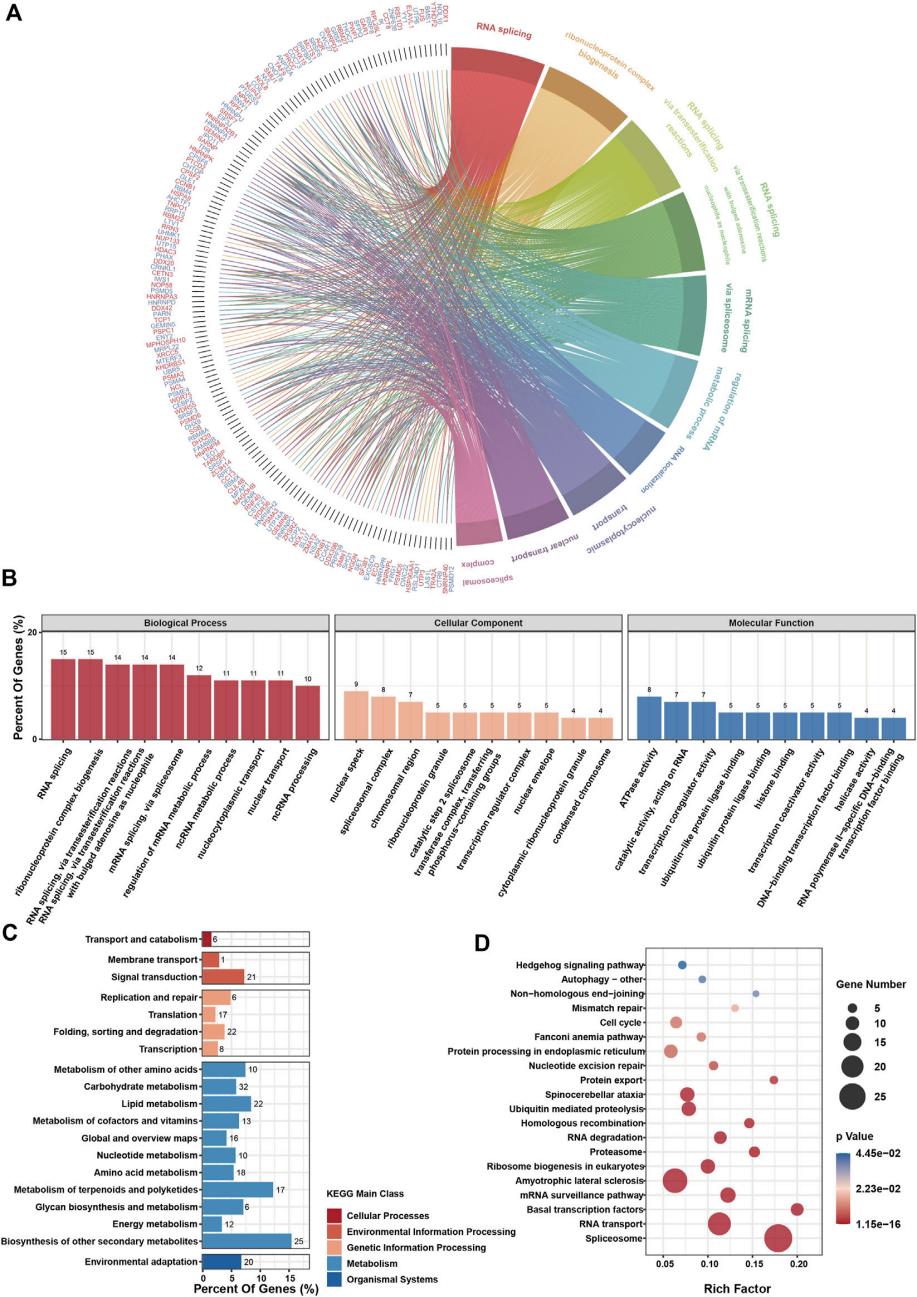
RIOK2-related genes enrichment analysis in tumor tissues.**(A)** The chord diagram depicts gene enrichment in the top ten biological processes processed by the GO enrichment analysis. **(B)** GO enrichment analysis of the top 500 RIOK2 expression-related genes in various types of tumor tissues. The functions of these genes were classified into three categories: biological processes, cellular components, and molecular functions. The ordinate represents the percentage of genes enriched for this function. **(C)** KEGG enrichment analysis of the top 500 RIOK2 expression-related genes in various types of tumor tissues. The functions of these genes enriched by KEGG are classified into five main classes: cellular processes, environmental information processing, genetic information processing, metabolism, and organismal systems. The ordinate represents the percentage of genes enriched in the function. **(D)** The ScatterPlot shows the KEGG enrichment pathways, which have significantly rich factors (*p* < 0.05).

To extract co-expressing genes, we calculated the Spearman correlation of the expression value against the RIOK2 gene in the expression matrix (Figure 8C). Genes that correlated with the expression of the RIOK2 with top 500 Spearman correlation coefficients were defined as genes co-expressing RIOK2. We then performed KEGG pathway and GO enrichment analyses of signaling pathways. There were two types of visualization to plot the gene co-expression data and display their functional annotations: first, a Circos plot to display gene co-expression and highlight co-expressing genes (Figure 9A); second, a functional histogram and bubble chart to display functional annotation of the co-expressing gene (Figures 9B–D). The results indicated that genes co-expressed with RIOK2 were involved in the processes of metabolism, cell cycle, and autophagy (Figure 9C). Furthermore, these genes were directly or indirectly associated with key molecules in crucial signaling pathways, namely the Hedgehog signaling pathway, Fanconi anemia pathway, and mRNA surveillance pathway (Figure 9D).

## Discussion

RIOK2 was discovered 12 years ago but has been the subject of only 17 publications since, resulting in a limited amount of information regarding its normal and oncogenic functions. The involvement of RIOK2 as an oncogenic driver was described for a spectrum of tumor types. Based on our novel conceptualization of RIOK2-mediated tumorigenesis and development, we performed a pan-cancer analysis based on multi-omics for a comprehensive panel of RIOK2 for the first time. A range of new and powerful bioinformatics tools revealed that RIOK2 is a candidate target across many cancer types which may stimulate novel ideas and strategies for the development of anti-neoplastic drugs. This is the first and most comprehensive analysis of the cancer-associated disruption of RIOK2.

To date, RIOK2 has been found to be highly expressed only in non-small cell lung cancer (Liu et al., 2016; Liu et al., 2018), prostate cancer (Read et al., 2013), glioblastoma (Liu et al., 2016), acute myeloid leukemia (Messling et al., 2021), and hepatocellular carcinoma cells (Delman et al., 2019). However, we showed that RIOK2 is common not only in these four cancer types but also in CHOL, DLBC, THYM, LGG, COAD, KIRC, HNSC, STAD, SKCM, and ESCA. Due to its prevalence in pan-cancer, we reasoned that RIOK2 may have profound clinical applications. As expected, the incidence of RIOK2 was relatively higher in metastatic SKCM, BRCA, KICH, COAD, KIRP, OV, ccRCC, LUAD, and UCEC and was lower in THCA than in primary tumors. Cox proportional hazards regression revealed an association between higher expression of RIOK2 and shorter overall survival and relapse-free survival. These findings reflect that more intensive testing for RIOK2 expression in pan-cancer can provide an earlier diagnosis and treatment with fewer cases of progression to metastatic cancer.

In addition to the effect on gene expression, RIOK2 can also be regulated at multiple levels, such as epigenetic modifications, gene alterations, and post-translational modifications. Changes in DNA methylation in cancer have been identified as promising targets for the development of robust diagnostic, prognostic, and predictive biomarkers (Koch et al., 2018). We observed that pan-cancer tumors had distinct DNA methylation profiles and that a negative correlation existed between DNA methylation levels in the promoter region of RIOK2 and RIOK2 expression levels. Hereby, we speculated that methylation regulation might participate in tumorigenesis and cancer development to affect patient survival. Thus, the potential of RIOK2 methylation assessments in cancer should be explored in the future.

Of note, although evidence concerning the association between gene alterations and cancer is mounting (Skoulidis and Heymach, 2019), no clinical data existed for the correlation between RIOK2 alterations and tumors. This study provides the first comprehensive report examining the association between tumor response and specific mutations in RIOK2. Among the six mutation sites examined, Ile235, which was mutated to Val235, was strongly associated with RIOK2 kinase activity. We further showed that the RIOK2 mutation was strongly and negatively correlated with patient survival in BRCA.

In addition, the accumulation of hyperphosphorylated kinase is also linked to cancer; however, the relationship between RIOK2 phosphorylation and tumors has not been reported in the literature. Our previous studies have shown that RIOK2 is phosphorylated by ATP activation at Asp257 in the active site, which could trigger late cytoplasmic 40S subunit biogenesis42. In this study, we showed for the first time that the protein kinase activity of RIOK2 was enhanced; six amino acids were identified to be phosphorylated frequently in pan-cancer, among which Ser149 and Asn397 appeared to be tumor-specific phosphorylation sites.

It remains unknown what role the aberrance in RIOK2 expression and function plays with immune cells in the tumor microenvironment. It is worth noting that the tumor microenvironment is one of the important factors leading to tumor migration and invasion (Kaymak et al., 2021). To better understand the relationship between RIOK2 and tumor-immune escape, we profiled and compared the changes in RIOK2 expression and tumor microenvironment immune cell composition in pan-cancer. We found that the infiltration levels of CD8+T cells, CD4+Th2 cells, CD4+Treg cells, and cancer-associated fibroblasts increased with elevated RIOK2 expression. As expected, we observed upregulation of known inhibitory immune checkpoints (CD274, IDO1, KDR, and transforming growth factor beta receptor I), markers of progressive CD8+T cell exhaustion (HAVCR2, ENTPD1, TIGIT, TNFRSF9, LAYN, PHLDA1, and SNAP47), and CD4+Th2/Th1, all of which demonstrated that RIOK2 might be a potential therapeutic target governing key hallmarks of immune suppression in various cancer types. Although RIOK2 has been found to be strongly correlated with tumor survival and metastasis, the underlying molecular mechanism requires further exploration. Recently, RIOK2 has been shown to be involved in tumor migration, invasion, and epithelial-mesenchymal transition through the AKT/mechanistic target of rapamycin kinase signaling pathway in glioblastoma and non-small cell lung cancer9; 10. Understanding the detailed mechanisms and co-occurrence genes of RIOK2 in various pathways is critical for the development of new therapeutic approaches that can improve patient care. Our results showed that five proteins (TSR1, NOB1, PNO1, LTV1, and bystin-like protein) among the 76 proteins we investigated interacted with RIOK2. Interestingly, it has been reported that NIN1 (RPN12) binding protein 1 homolog can determine where in the spectrum of chronic myeloid leukemia progression an individual patient should be diagnosed (Oehler et al., 2009). The partner of the NOB1 homolog is also overexpressed in colorectal cancer and correlates with poor patient survival, and it exerts oncogenic effects by altering ribosome biogenesis (Shen et al., 2019). Bystin-like protein plays an important role in the rapid growth of hepatocellular carcinoma cells, mediates nucleolus-derived foci and prenucleolar body formation, and ultimately contributes to nucleolar assembly during cell division (Wang et al., 2009). Therefore, we reasoned that it is more likely that it is through these three proteins that RIOK2 contributes to the survival, proliferation, and invasion of cancer cells in pan-cancer. In addition, enrichment analysis of the top 500 genes related to RIOK2 revealed that multiple pathways were correlated with metabolism, cell cycle, and autophagy. Among the above signaling pathways, Hh and autophagy signaling pathways play critical roles in cancer stem cell progression (Yang et al., 2020). Fanconi anemia is a genetic disorder characterized by predisposition to cancer. Carcinogenesis resulting from a dysregulated Fanconi anemia pathway is multifaceted, as Fanconi anemia proteins monitor multiple complementary genome surveillance checkpoints throughout the interphase (Nalepa and Clapp, 2018). In addition, dysregulated cell cycle transition caused by inefficient proteolytic control leads to uncontrolled cell proliferation and finally results in tumorigenesis (Dang et al., 2021). Overall, RIOK2 may be a critical factor in the occurrence and development of tumors by regulating these pathways. However, further studies are required to validate these assumptions.

An important merit of this study is the comprehensive understanding of RIOK2 from multiple perspectives, which can provide guidance for future research. Our findings demonstrate that RIOK2 expression, methylation, alteration, and phosphorylation play essential roles in tumor occurrence and metastasis, which not only reveals a novel target of RIOK2-mediated proliferation, migration, and invasion of tumor cells, but also provides a basis for treating malignant tumors by interfering with RIOK2.

## Conclusion

Existing evidence has shown that RIOK2 is involved in non-small cell lung cancer, glioblastoma, hepatocellular carcinoma, acute myeloid leukemia, and prostate cancer progression. However, the role of RIOK2 in tumors remains unclear. Therefore, it is essential to determine whether RIOK2 can be used as a predictive biomarker for a broader range of tumor types. In this study, for the first time, we found that elevated RIOK2 expression, methylation, mutation and hyper-phosphorylation were surprisingly common across multiple cancer types. In conclusion, this study is the first comprehensive analysis of multi-omic features of RIOK2 across pan-cancer and helps in unraveling the role of RIOK2 in cancer. After further functional validation, RIOK2 may be utilized as a cancer biomarker which can be utilized to implement novel targeted therapies for a range of tumors.

## Data Availability

The datasets presented in this study can be found in online repositories. The names of the repository/repositories and accession number(s) can be found in the article/Supplementary Material.
