# Supplementary figures and images for: Pan-cancer analyses reveal multi-omics and clinical characteristics of RIO kinase 2 in cancer
Source: Front Chem. 2022 Nov 28;10:1024670. doi: 10.3389/fchem.2022.1024670 (PMC9742535; doi:10.3389/fchem.2022.1024670)

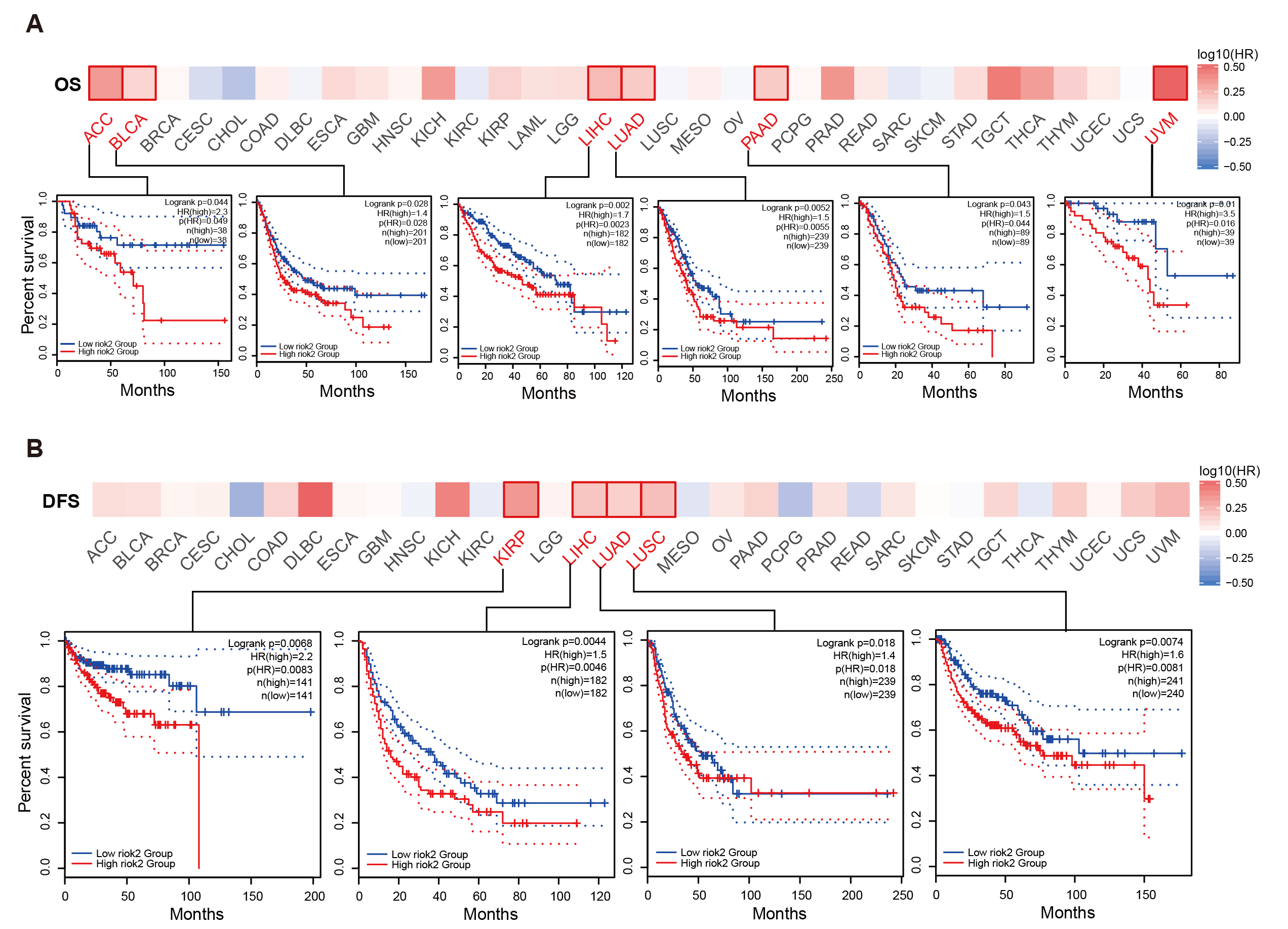

Supplement: Supplementary file 1 [file Image3.TIF]

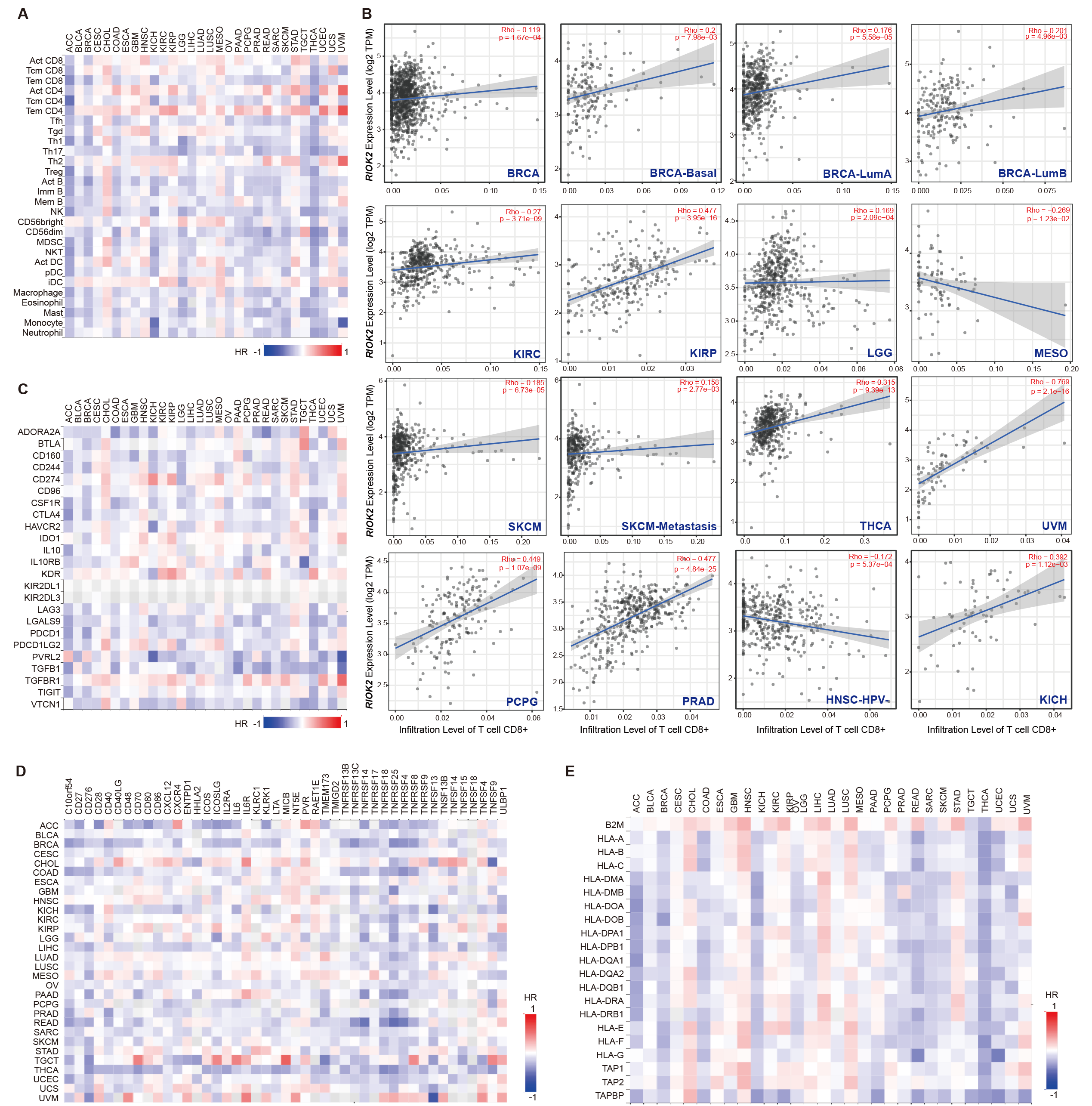

Supplement: Supplementary file 2 [file Image4.TIF]

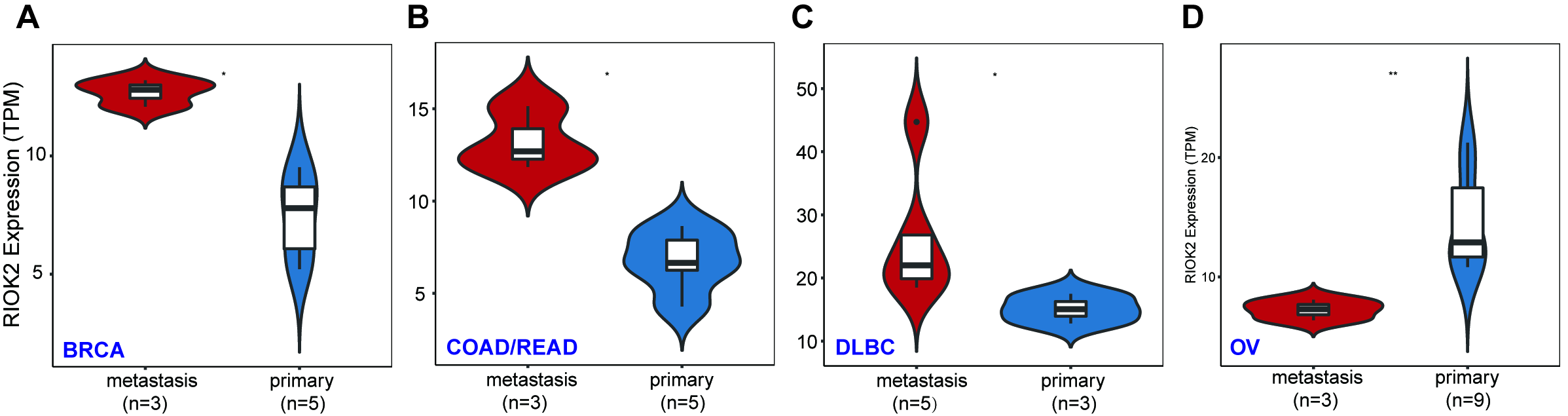

Supplement: Supplementary file 4 [file Image2.TIF]

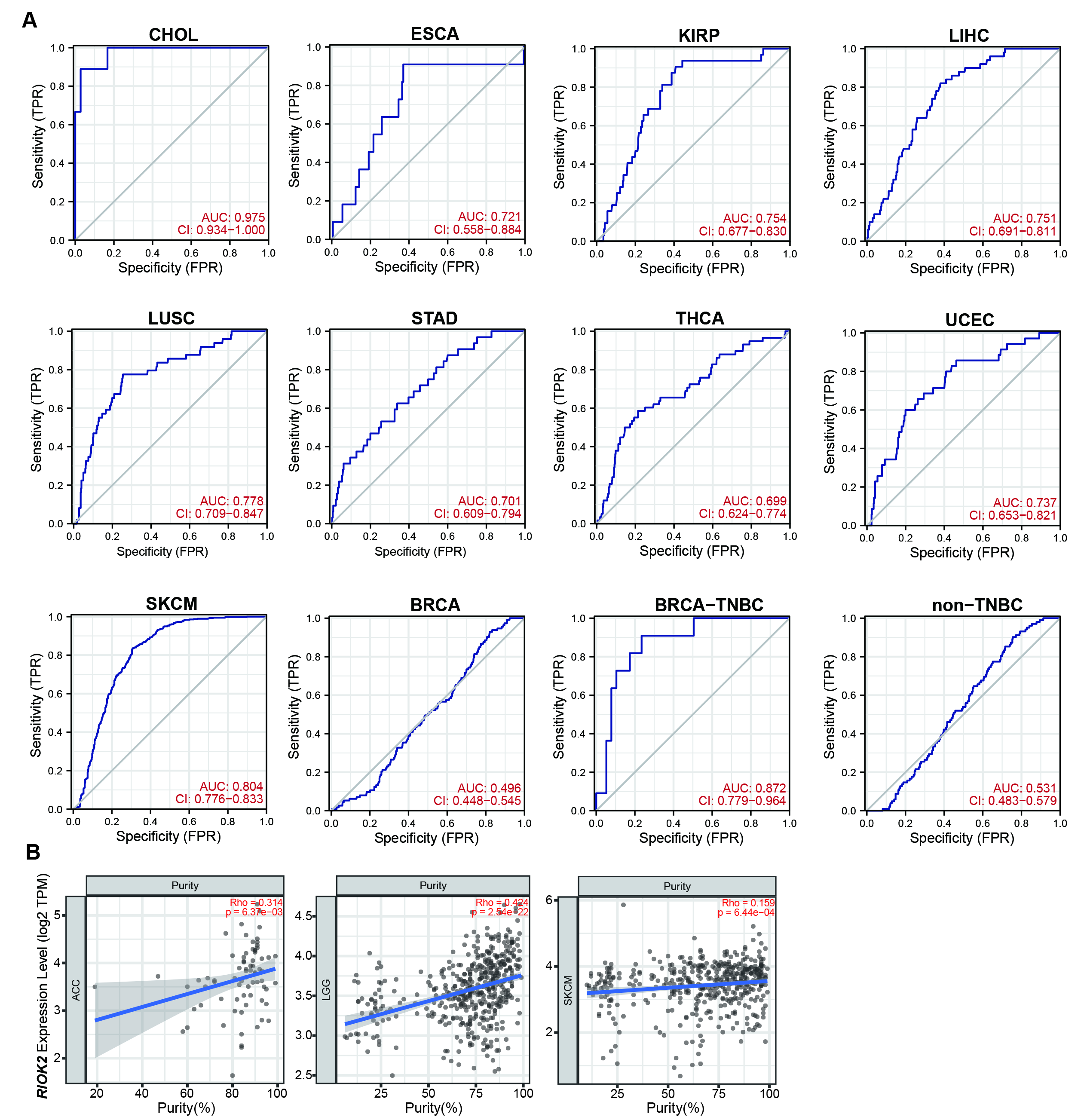

Supplement: Supplementary file 5 [file Image1.TIF]

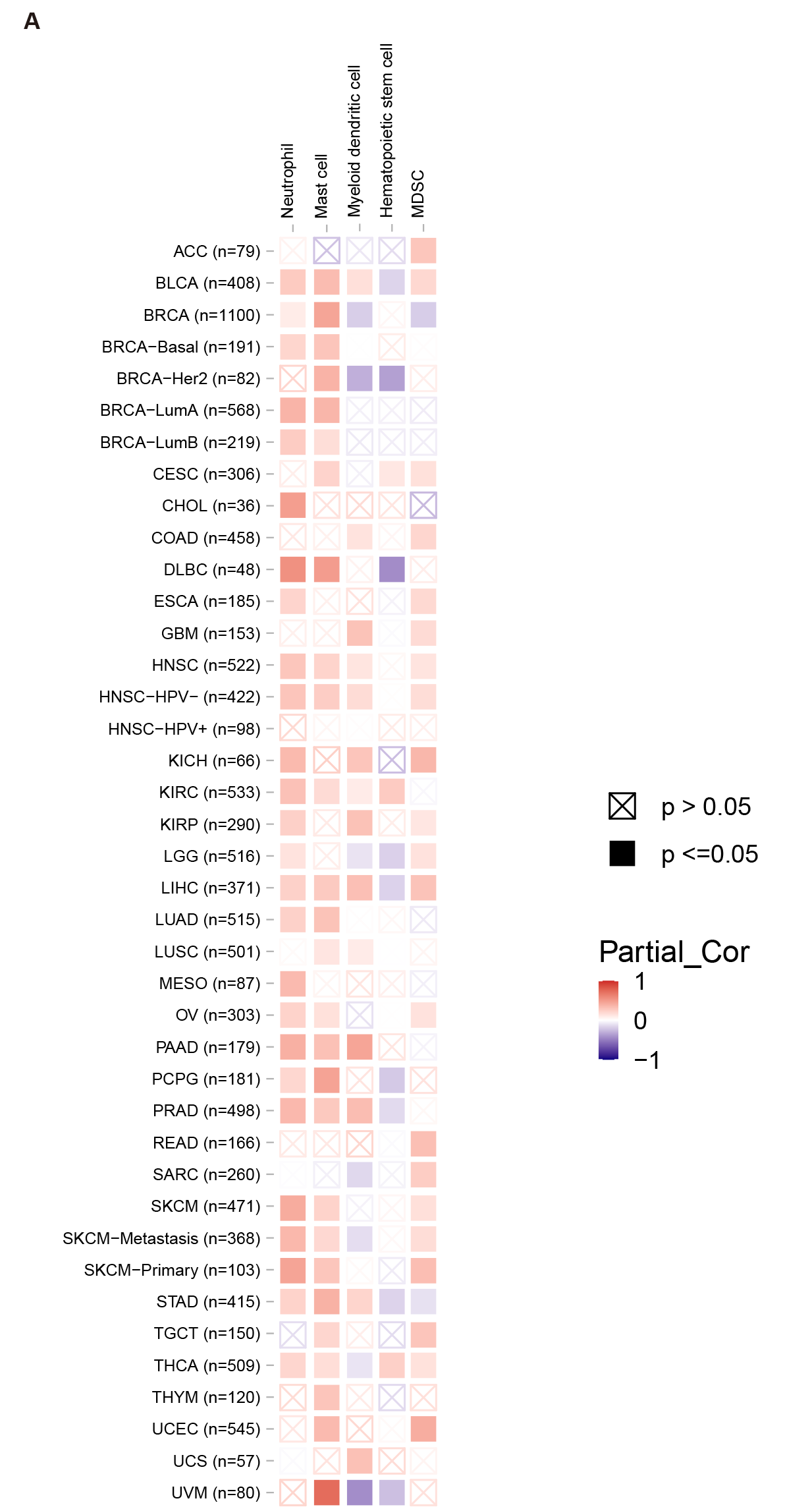

Supplement: Supplementary file 7 [file Image5.TIF]
