# Supplementary material for: Pan-cancer analyses reveal multi-omics and clinical characteristics of RIO kinase 2 in cancer
Source: Front Chem. 2022 Nov 28;10:1024670. doi: 10.3389/fchem.2022.1024670 (PMC9742535; doi:10.3389/fchem.2022.1024670)
Supplement: Supplementary file 6 [file DataSheet2.docx]

**GO&KEGG Rscript**

library(GOplot)

library(stringr)

library(org.Hs.eg.db)

library(clusterProfiler)

#KEGG

gene_symbol <- read.table(file = "500_table_similar.txt", header=T)$GeneSymbol

gene <- mapIds(org.Hs.eg.db, gene_symbol, 'ENTREZID', 'SYMBOL')

kk <- enrichKEGG(gene = gene, organism ="human",

pvalueCutoff = 0.05,

qvalueCutoff = 0.01,

minGSSize = 1,

use_internal_data =FALSE)

write.csv(as.data.frame(kk@result), file="enrichKEGG.csv")

dotplot(kk,showCategory=30)

barplot(kk,showCategory=30,drop=T,title = "EnrichmentKEGG",font.size = 10 )

#GO

entrez_id <- bitr(gene_symbol, fromType = "SYMBOL",toType = c("ENTREZID","ENSEMBL"),OrgDb = org.Hs.eg.db)

ego_all <- enrichGO(gene = entrez_id$ENTREZID,OrgDb = org.Hs.eg.db,ont = "ALL",pAdjustMethod = "BH",pvalueCutoff = 0.05,readable = TRUE,pool = TRUE)

GO=ego_all[1:10,c(1,2,3,9,7)]

GO$geneID=str_replace_all(GO$geneID,"/",",")

names(GO)=c("Category","ID","term","Genes","adj_pval")

gene=data.frame(ID=gene_symbol,logFC=rnorm(length(gene_symbol),mean = 0,sd=2))

circ<-circle_dat(GO,gene)

chord<-chord_dat(data = circ,genes = gene,process = GO$term)

GOChord(chord,space = 0.02,gene.order = 'logFC',gene.space = 0.25,gene.size = 5)

out_name="binfohome_goplot_all"

cairo_pdf(file=paste(out_name,"_GOChord.pdf",sep = " "),onefile = TRUE,width = 22,height = 20)

GOChord(chord,space=0.02,gene.order = 'logFC',gene.space = 0.25,gene.size = 5)

dev.off()

write.csv(as.data.frame(ego_all), file="enrichGO.csv")

dotplot(ego_all,showCategory=30)

barplot(ego_all,showCategory=30,drop=T,title = "EnrichmentGO",font.size = 10 )

**ROC Rscript**

cancer_type<-"TCGA-THCA"

data_type<-"Gene Expression Quantification"

data_category<-"Transcriptome Profiling"

workflow_type<-"HTSeq - Counts"

query_TranscriptomeCounts<-GDCquery(project=cancer_type,data.category=data_category,data.type=data_type,workflow.type=workflow_type)

GDCdownload(query_TranscriptomeCounts,method="api")

expdat<-GDCprepare(query=query_TranscriptomeCounts)

count_matrix=assay(expdat)

group_list_1<-ifelse(as.numeric(str_sub(colnames(count_matrix),14,15))<10,"tumor","normal")

normal<-count_matrix[,which(group_list_1=="normal")]

tumor<-count_matrix[,which(group_list_1=="tumor")]

which(rownames(tumor)=="ENSG00000058729")

which(rownames(normal)=="ENSG00000058729")

RIOK2_KICH_tumor<-tumor[792,]

RIOK2_KICH_normal<-normal[792,]

write.csv(RIOK2_KICH_tumor,file = "RIOK2_STAD_tumor.csv")

write.csv(RIOK2_KICH_normal,file = "RIOK2_STAD_normal.csv")

**TCGA&GTEx analysis Rscript**

rm(list=ls())

options(stringsAsFactors = F)

setwd("F:/GTEX TCGA 提取表达矩阵")

#数据来源

#TcgaTargetGtex_rsem_gene_fpkm https://xenabrowser.net/datapages/?dataset=TcgaTargetGtex_rsem_gene_fpkm&host=https%3A%2F%2Ftoil.xenahubs.net&removeHub=https%3A%2F%2Fxena.treehouse.gi.ucsc.edu%3A443

#TcgaTargetGTEX_phenotype.txt https://xenabrowser.net/datapages/?dataset=TcgaTargetGTEX_phenotype.txt&host=https%3A%2F%2Ftoil.xenahubs.net&removeHub=https%3A%2F%2Fxena.treehouse.gi.ucsc.edu%3A443

####提取UCEC Xena####

library(data.table)

library(org.Hs.eg.db)

library(stringr)

####读入表型文件####

pd<-fread("TcgaTargetGTEX_phenotype.txt.gz")

pd<-as.data.frame.matrix(pd)

table(pd[,4])

pd_Lung<-pd[pd[,4]=="Adrenal gland",]

#提取GTEX全部（包括cell）

pd_GTEX<-pd_Lung[pd_Lung[,7]=="GTEX",]

pd_gtex<-as.character(pd_GTEX$sample)

#提取TCGA

pd_TCGA<-pd_Lung[pd_Lung[,7]=="TCGA",]

pd_tcga<-as.character(pd_TCGA$sample)

#提取LUSC

pd_TCGA_LUSC<-pd_TCGA[pd_TCGA[,3]=="Adrenocortical Cancer",]

pd_tcga_LUSC<-as.character(pd_TCGA_LUSC$sample)

#区分癌旁和肿瘤

pd_TCGA_type_LUSC<-ifelse(as.numeric(str_sub(pd_TCGA_LUSC[,1],14,15))<10,"tumor","normal")

#肿瘤组织样品

pd_TCGA_TUMOR_LUSC<-pd_TCGA_LUSC[pd_TCGA_type_LUSC=="tumor",]

pd_tcga_tumor_LUSC<-as.character(pd_TCGA_TUMOR_LUSC$sample)

#癌旁组织样品

pd_TCGA_NORMAL_LUSC<-pd_TCGA[pd_TCGA_type_LUSC=="normal",]

pd_tcga_normal_LUSC<-as.character(pd_TCGA_NORMAL_LUSC$sample)

####读入表达矩阵文件####

exp<-fread("TcgaTargetGtex_rsem_gene_tpm.gz")

exp<-as.data.frame.matrix(exp)

#表达矩阵预处理

exp[1:5,1:5]

rownames(exp)<-exp[,1]

exp[1:5,1:5]

exp<-exp[,2:ncol(exp)]

#提取RIOK2

which(rownames(exp)=="ENSG00000112578.1")

RIOK2_TCGA_GTEX<-exp[34149,]

##输出

#GTEX

exp_GTEX<-RIOK2_TCGA_GTEX[,colnames(RIOK2_TCGA_GTEX)%in%pd_gtex]

write.csv(t(exp_GTEX),file = "TPM-RIOK2-GTEX.csv")

#TCGA_NORMAL

exp_TCGA_LUSC<-RIOK2_TCGA_GTEX[,colnames(RIOK2_TCGA_GTEX)%in%pd_tcga_normal_LUSC]

write.csv(t(exp_TCGA_LUSC),file = "TPM-RIOK2-TCGA-NORMAL-LUSC.csv")

#TCGA_TUMOR

exp_TCGA_TUMOR_LUSC<-RIOK2_TCGA_GTEX[,colnames(RIOK2_TCGA_GTEX)%in%pd_tcga_tumor_LUSC]

write.csv(t(exp_TCGA_TUMOR_LUSC),file = "TPM-RIOK2-TCGA-TUMOR-LUSC.csv")
