# Supplementary material for: Pan-cancer analyses reveal multi-omics and clinical characteristics of RIO kinase 2 in cancer
Source: Front Chem. 2022 Nov 28;10:1024670. doi: 10.3389/fchem.2022.1024670 (PMC9742535; doi:10.3389/fchem.2022.1024670)
Supplement: Supplementary file 8 [file DataSheet1.docx]

**Supplementary Figure Legends**

**Supplemental Figure 1. Expression level of *RIOK2* gene in tumor cells and evaluation of RIOK2 as a diagnostic marker , related to Figure 1.**

1. Summarized ROC curves and diagnostic performance of RIOK2 by AUROC estimated for all patients in pan-cancer.
2. The relationship between RIOK2 expression and tumor purity was used to evaluate the difference in RIOK2 expression between cancer cells and other infiltrating cells in the tumor micro-environment. The correlation of RIOK2 expression and tumor purity were visualized by scatter plots using the partial Spearman correlation with p-value<0.05.

**Supplemental Figure 2. Expression level of *RIOK2* gene in primary and metastatic tumor cell lines**

(A-D) The difference in RIOK2 expression between primary and metastatic tumor cell lines. The blue violin represents the healthy group, and the red one represents the tumor group. Statistical significance, as computed using the Wilcoxon test, is annotated by the number of stars (* p < 0.05, ** p < 0.01, *** p < 0.001, **** p < 0.0001).

**Supplemental Figure 3, related to Figure 3.**

(A-B) The heat maps convey the correlation between the RIOK2 expression and OS and RFS across multiple cancer types using a univariate Cox analysis with split expression percentage of 50 % patients. The bold border of the heatmap represents the high hazard ratio (HR) with p-value<0.05. The OS and RFS Kaplan-Meier curves of the various cancer types which have significant survival risk (p-value<0.05) were displayed.

**Supplemental Figure 4, related to Figure 7.**

The heatmap conveys the correlation of *RIOK2* expression with (A) tumor-infiltrating lymphocytes, (C) immune checkpoints, (D) immune stimulators and (E)MHC moleculars in diverse cancer types. (B)Scatter plots present the relationship between CD8^+^T cells infiltrates estimation value and RIOK2 expression. We employed the Spearman's correlation to perform purity adjustment analysis.

**Supplemental Figure 5. Expression level of *RIOK2* gene in other immune cells**

1. A heat map showing the correlation between RIOK2 expression and immune infiltration levels in diverse cancer types. The correlation of infiltrating cells were obtained using the EPIC algorithm.
